# *Schizonepeta Tenuifolia* with *Alpinia Oxyphylla* Alleviates Atopic Dermatitis and Improves the Gut Microbiome in Nc/Nga Mice

**DOI:** 10.3390/pharmaceutics12080722

**Published:** 2020-07-31

**Authors:** Ting Zhang, Jingyi Qiu, Xuangao Wu, Shaokai Huang, Heng Yuan, Sunmin Park

**Affiliations:** Department of Food and Nutrition, Obesity/Diabetes Research Center, Hoseo University, Asan 31499, Korea; zhangting92925@gmail.com (T.Z.); yoco2464@gmail.com (J.Q.); niyani0@naver.com (X.W.); huangsk0606@gmail.com (S.H.); yuanheng.changan@gmail.com (H.Y.)

**Keywords:** atopic dermatitis, gut microbes, Nc/Nga mice, *Schizonepeta tenuifolia* Briquet, *Alpinia oxyphylla* Miquel

## Abstract

Atopic dermatitis (AD) is a chronic inflammatory skin disease that may be related to gut microbes. *Schizonepeta Tenuifolia* Briquet (STB) and *Alpinia Oxyphylla* Miquel (AOM) has traditionally been used for anti-inflammatory activity. We evaluated the effects of STB, AOM and STB+AOM extracts on 2,4-dinitro-1-chlorobenzene (DNCB)-induced AD skin lesions in Nc/Nga mice and action mechanism was explored. AD lesions were induced in the dorsal skin of Nc/Nga mice by topical application of 1% followed by 0.2% DNCB. After DNCB was applied, the mice had topical applications of either 30% water, 0.01% dexamethasone, 30% STB, 30% AOM, 15% STB + 15% AOM extracts in butylene glycol (BG). Each group was also fed corresponding high-fat diets with 1% dextrin (AD-Con and AD-Positive), 1% STB (AD-STB), 1% AOM (AD-AOM) and 0.5% STB + 0.5% (AD-MIX). Normal-control mice had no DNCB application. The study evaluated the skin AD severity, scratching behavior and weight changes of AD mice for 5 weeks. Compared with AD-Con, AD-STB, AD-AOM and AD-MIX alleviated the clinical AD symptoms (erythema, pruritus, edema, erosion and lichenification and scratching behaviors), normalized immune chemistry (serum IgE concentration, mast cells and eosinophil infiltration), improved skin hyperplasia and enhanced the gut microbiome. AD-STB, AD-AOM, AD-MIX and AD-positive treatments inhibited cutaneous mRNA expression of TNF-α, IL-4 and IL-13 and serum IgE concentrations. AD-MIX most effectively reduced clinical AD symptoms and proinflammatory cytokines. AD-Positive also reduced them but serum GOT and GPT concentrations were abnormally high. AD-STB and AD-MIX increased the alpha-diversity of fecal bacteria and reduced the serum acetate concentration, compared to the AD-Con. In conclusion, the mixture of STB and AOM is effective for treating AD symptoms locally and systemically without adverse effects and are potential interventions for atopic dermatitis.

## 1. Introduction

Atopic dermatitis (AD) is a complex, chronic, recurrent inflammatory disease [1,2]. Its etiology involves interactions between the internal immune system and external factors [1,2]. About 60% of AD children may have one or more atopic syndromes including allergic rhinitis, food allergies and asthma [3]. This characteristic disease progression is known as the “atopic march” and its pathophysiology is the subject on-going study, although it is involved in over-activation of immunity [4]. However, the etiology of atopic dermatitis remains unclear. AD results in the epidermal barrier dysfunction and overactivation of the immune response to increase inflammation when exposed to allergens. Antigens including mites stimulate skin dendritic cells and trigger Th2 lymphocyte activation to release pro-inflammatory cytokines interleukin (IL)-4 and IL-13 [1,4,5,6]. Eosinophils and mast cells contribute to IL-4 and IL-13 production and release that induces serum immunoglobulin E (IgE) concentrations [1,4,5,6]. As a result, AD induces severe itching, dry skin, lichenification, eczema and edema by damaging skin barriers and inducing persistent inflammation [2,4,7]. However, there are no effective interventions that address the fundamental causes of AD. Topical and/or oral corticosteroids are effective first-line drugs for the treatment of AD but they are prone to dependence and cause adverse effects such as telangiectasia, tingling and skin atrophy [8]. Immunomodulators such as tacrolimus and pimecrolimus creams are also used for AD therapy [7].

Recent research indicates that intestinal microflora disturbances are closely related to AD incidence [5,9]. The large intestines are the largest “bacteria depot” in the body and gut microbiota participates in regulating the systemic inflammatory response through the modulation of the production and/or removal of endotoxins and short-chain fatty acids (SCFA) [5,10]. It takes a long time to establish the eubiosis in the intestinal mucosa with a balanced ecological environment and it is difficult to return a state of dysbiosis to gut microbiome eubiosis. Eubiosis gives the intestines the ability to recover and maintain stability and resists the invasion and overgrowth of external pathogens [11]. Intestinal bacteria produce various metabolites including SCFAs, neurotransmitters, inflammatory cytokines and endotoxins that affect the host metabolism, especially immune function [12,13]. The specific mechanism of intestinal flora affecting AD is still unclear but dysbiosis of the gut microbiome is involved in AD etiology [14]. Propionic acid has anti-inflammatory properties by inhibiting Th2 and Th17 cell differentiation and proliferation to reduce to decrease the expression of TNF-α, IL-10 and macrophage markers, thereby suppressing allergic diseases [13]. Herbal extracts contain soluble dietary fibers to be converted into SCFA including propionate and butanoate and they also include phytochemicals to modulate gut microbiome composition. Therefore, the eubiosis of the gut microbiome protects against and alleviates AD symptoms.

In China, Korea and Japan, traditional Chinese medicine is commonly used as a safe and effective treatment for AD [15]. The Chinese medicine is mainly made of hot water extraction of herbs and their phytochemicals are mostly extracted. *Schizonepetae Spica (Schizonepeta tenuifolia* Briquet or *Nepeta tenuifolia* Benth) is a *Labiatae* plant and its aerial part, including flowers and stems, is used for medication. It mainly contains essential oil such as 47.7% l-pulegone, 14.3% d-menthone, 5.4% schizonal, 4.1% cis-pulegone oxide, 1.9% piperitone [16,17]. It is classified as a relieving agent of the skin disorders including eczema and psoriasis, in traditional Chinese medicine and it has been also used for the treatment of colds, fevers and inflammation and its anti-microbial and anti-viral activities have been reported in vitro and in vivo studies [18]. *Alpinae oxyphyllae* fructus (*Alpinia oxyphylla* Miquel) is the fruit of the Zingiberaceae (ginger) plant. It is reported to contain about 85 compounds with 61.2% sesquiterpenoids, 9.4% monoterpenes, 6.0% 2-hydroxy-2-isopropenyl-5-methy-cyclohexanone, 5.9% dipheyl-heptanes, 7.1% flavonoids, 2.0% limonene, and, 1.2% diterpenoids [19,20]. Its pharmacological activities mainly include anti-tumor, anti-aging, neuronal protective, anti-inflammatory and anti-allergy activities and it has been used as herbal medicine [19,20].

The pathology of the skin lesions and immunological alterations of Nc/Nga mice are similar to human atopic dermatitis and they are commonly used animal models for AD research [21]. Nc/Nga mice will spontaneously develop AD-like lesions and exhibit the characteristic elevation of plasma IgE concentrations but do not induce the skin lesion coverage over a normal circumference [7]. Local application of 2,4-dinitro-1-chlorobenzene (DNCB) can initiate the development of AD-like skin lesions in Nc/Nga mice in a defined region [22]. Since not only the local application of herbal extract but also its intake will alleviate AD symptoms [23], we hypothesized that hot water extracts of *Schizonepetae Spica* and *Alpinae oxyphyllae* fructus could improve DNCB-induced AD lesions in Nc/Nga mice. The hypothesis was examined and the action mechanism was explored. This is the first study to demonstrate that the intake and the local application of these two herbs mixture alleviate AD symptoms, to the best of our knowledge.

## 2. Materials and Methods

### 2.1. Preparation of Schizonepetae Spica and Alpinae Oxyphyllae Fructus Water and 1,3-Butylene Glycol Extracts

The aerial part of *Schizonepeta tenuifolia* Briquet including flower and stem and the fruit of *Alpinia oxyphylla* Miquel was purchased from Kyung-Dong Herbal market (Seoul, Korea) and their authenticity was confirmed by Dr. Young Seng Joo (Woo Suk University, Jeonju, Korea) in 2018. Herbs were provided by oral intake and local application in the dorsal skin. Hot water extracts of herbs have been traditionally used for the treatments for diseases and water extracts of *Schizonepeta tenuifolia* Briquet and *Alpinia oxyphylla* Miquel were prepared for oral intake for AD in this study. Each herb (1 kg) was boiled in water (10 L) at 95 °C for 3 h, filtered and the filtrates evaporated in a rotary evaporator. The concentrates were then lyophilized and used for oral intake. The yields of *Schizonepeta tenuifolia* Briquet and *Alpinia oxyphylla* Miquel water extracts were 18.8 and 25.9% based on each dried herb added.

Since 1,3-butylene glycol is one of the major solvents for cosmetic usages, it was used as the solvent to make the herbal application for the dorsal skin. Herbal lotion for the dorsal skin was made with 30 g *Schizonepetae Spica* or *Alpinae oxyphyllae* dried powder in 100 mL 1,3-butylene glycol (BG; Junsei Chemical, Chuo-ku, Japan) at 60 °C in the ultrasonic chamber (Lab Companion; Daejeon, Korea) for 4 h. The 30% BG extract was filtered and the filtrate was used for application on the dorsal skin. Total phenolic compounds in each herbal extract were measured by Folin–Ciocalteu reagent and the amounts were expressed as mg gallic acid equivalents·g^−1^ [24]. Total flavonoids were measured using a modified method published by Davis [25,26] and rutin was used as the standard.

### 2.2. Experimental Animal

Sixty male Nc/Nga mice aged 7–8 weeks (18–26 g) were purchased from Dae Han Bio Link (Um-Sung, Korea). They were acclimated in our animal facility with a temperature of 23 ± 3 °C, a humidity of 50 ± 10% and 12 h of light (08: 00–20: 00) and dark (20: 00–08: 00) for 1 week. The mice were freely provided food and water in individual cages. All research protocols followed the guidelines for the National Institute of Health and Animal Care and were approved by the Animal Care and Use Review Committee of Hoseo University (Asan, Korea; HSIACUC-18-230; approved on 06-01-19).

Nc/Nga mice were anesthetized with a mixture of ketamine and xylazine (100 mg 10 mg /kg body weight, respectively; Bayer AG, Leverkusen, Germany). Hairs on the back of the Nc/Nga mice were completely removed and DNCB (Sigma Co., St. Loise, MO, USA) was applied to the dorsal skin to induce AD lesions. On the first day, 1% DNCB (200 µL) in the solvent (acetone:Olive oil = 3:1) was applied. After the first application of 1% DNCB, 0.2% DNCB (100 µL) was applied every other day for 3 weeks. All mice were provided with a high-fat diet (43% fat diet) to exacerbate AD symptoms.

### 2.3. Experimental Design

**Figure 1:**
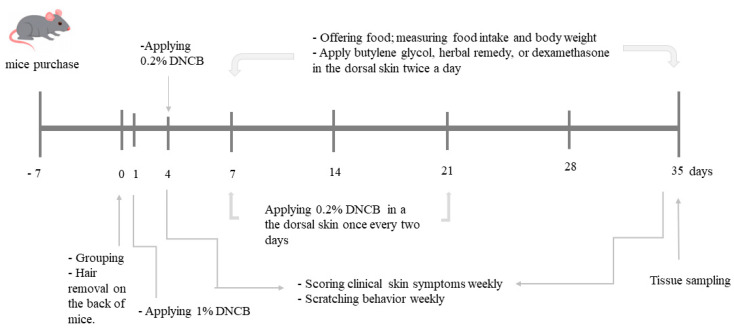
Experimental design.

AD lesion-induced Nc/Nga mice were divided into five groups: 1) dextrin (AD-Con), 2) dexamethasone (AD-Positive), 3) *Schizonepetae Spica* (AD-STB), 4) *Alpinae oxyphyllae* (AD-AOM) and 5) *Schizonepetae Spica* + *Alpinae oxyphyllae* (AD-MIX). The Normal-Con mice, without DNCB-application, had an application of acetone and olive mixture on the dorsal skin and 1% dextrin containing diet. The mice had a local application on the dorsal skin and intake of assigned herbal extracts daily. After the 1st application of 1% DNCB, 200µl of BG (AD-Con), 0.01% dexamethasone (AD-Positive), 30% STB, 30% AOM, 15% STB + 15% AOM lotions were applied locally to the dorsal skin lesion of AD mice in the morning and evening. Since in the manual of topical corticosteroids to treat AD, dexamethasone is the first-line of AD treatment with mild potency, it was used as a positive control. In an animal study, 0.01% dexamethasone was used for topical application [26]. A high-fat diet was provided with 1% dextrin, 1% *Schizonepetae Spica* lyophilized powder, 1% *Alpinae oxyphyllae* lyophilized powder or 0.5% *Schizonepetae Spica* lyophilized powder plus 0.5% *Alpinae oxyphyllae* lyophilized powder for the assigned groups. Respective diets and water were freely provided from the first day of applying 0.2% DNCB. Food intake and body weight were measured weekly. The experimental schedule is presented in (Figure 1).

### 2.4. Evaluation of the Degree of Skin Damage and Scratch Behavior in AD Mice

Clinical evaluations of the dorsal skin and scratch behavior evaluation were conducted weekly in AD mice. Pictures of the dorsal skin lesions were taken and independently scored for the severity of clinical symptoms in the mice. The severity of clinical AD symptoms was blindly scored with the designated trained technician each week [27]. The severities of erythema, pruritus and dry skin, edema and excoriation, erosion and lichenification were scored. The rating scales were 0 points (asymptomatic), 1 point (mild), 2 points (moderate) and 3 points (severe) [28].

Scratching behavior was evaluated in AD mice of each group. Each AD mouse was placed in a transparent plastic cage and visually assessed for the number of scratch behaviors in every AD mouse for 10 min, including the front and back paws (ears, face, back) scratching behavior [29]. If AD mice scratched more than three times in a row, it was counted twice. If the AD mice kept scratching, the technician artificially intervened to cause the AD mouse to stop the scratching behavior. In the morning (10:00–11:00) 0.2% DNCB 100 µL was applied externally to induce skin lesions. The number of scratches was counted. Two hours later, after drug intervention and at noon the next day (12:00–13:00), the number of scratches was counted.

### 2.5. Sample Collection and Serum Analysis

At the end of the 5-week treatment, mice were anesthetized with a mixture of ketamine and xylazine (100 mg and 10 mg/kg body weight, respectively). The venous blood was taken from the inferior vena cava and then the liver and epididymal fat of the mice were collected and weighed using a precision balance scale (Sartorius, Germany). Serum was separated from the collected venous blood by centrifugation at 500 xg and the isolated organs were quickly put into liquid nitrogen and rapidly frozen. The feces were collected from the cecum of mice. Samples were stored for subsequent biochemical experiments in a freezer at −70 °C.

Total serum IgE was quantified by Enzyme-linked immunosorbent assay (ELISA kit II) (BD Biosciences, San Diego, CA, USA). according to the kit instructions. Serum glutamate oxaloacetate transferase (GOT), glutamate pyruvate transferase (GPT) and triglyceride concentrations were determined by colorimetry kits (Asan Pharm. CO, LTD, Seoul, Korea). The thiobarbituric acid reactive substances (TBARS) kit (Cayman Chemical, Ann Arbor, MI, USA) was used to measure lipid peroxide concentrations of skin tissues.

### 2.6. Histopathological Analysis

The large intestine and the dorsal skin tissues were fixed in 10% formalin (Sigma-Aldrich, St. Loise, MO, USA) and they were dehydrated with xylene and ethanol. The dehydrated tissues were embedded with paraffin. The sections (5μm) of the paraffin-embedded tissues were prepared by using a microtome (Leica Microsystems, Wetzlar, Germany) [30]. Hematoxylin & eosin (H-E; Sigma-Aldrich, St. Loise, MO, USA) staining was conducted to measure epidermis and dermis thickness, abnormality of cells and nucleus in the keratocytes and the surface of the skin. The intestinal histology of the large intestines (villous length, intestinal crypt, the width of intestinal villi) was evaluated in H-E staining [31]. Toluidine blue (Sigma-Aldrich, St. Loise, MO, USA) staining was also performed to calculate the number of mast cells in the tissues (%). H-E staining of the large intestine tissues was used to observe the histopathological changes. Using Alcian Blue-Periodic acid (AB-PAS) staining, the amount of mucin (%) was measured in the intestines. The above observations were magnified 100/200 times using an optical microscope (Axio Imager 2; Carl Zeiss AG, Oberkochen, Germany) and evaluated using I-Solution software including counter format to view histopathological changes. The thickness of dermis and epidermis was measured in the H-E stained section of the dorsal skin using I-Solution software.

### 2.7. Relative mRNA Expression of Skin Tumor Necrosis Factor (TNF)-α, IL-4 and IL-13

Total RNA was isolated from skin tissue using Trizol Reagent (Ambion Inc., Austin, TX, USA) reagent. The amount and purity of total RNA were assessed using a spectrometer 260 nm and 280 nm (Perkin Elmer, Boston, MA, USA). Each total RNA sample (1 µg) was used for synthesizing cDNA using the Superscript III reverse transcriptase kit (Bio-Rad, Richmond, CA, USA) that synthesizes each equal amount of total RNA into cDNA. The cDNA was mixed with SYBR Green supermix (Bio-Rad, Richmond, CA, USA) mixture and then amplified using CFX Connect™ Real-Time PCR Detection System (Bio-Rad Laboratories, Inc, Hercules, CA, USA). To evaluate the relative mRNA expression of the gene of interest in the skin using the Ct comparison method, the expression level of the genes was normalized to the expression of the housekeeping gene β-actin. Use the following primers designed and synthesized in the house for PCR reactions, mouse β-actin forward 5′-TCCTGTGGCATCCACGAAA CT-3′ and reverse 5′-GAAGCATTTGCGGTGGACGAT-3′. IL-4, forward 5′-GAATGTACCAGG AGCCATATC-3′ and reverse 5′-CTCAGTACTACGAGTAATCCA-3′. IL-13, forward 5′- CAGCT CCCTGGTTCTCTCAC-3′and reverse5′-CCACACTCCATACCATGCTG-3′. TNF-α, forward 5′-CCTGTAGCCCACGTCGTAGC-3′ and reverse5′-TTGACCTCAGCGCTGAGTTG-3′. The relative expression of each gene was evaluated by the Ct method [32].

### 2.8. Determination of Serum SCFA Concentrations

Serum SCFA concentrations were detected by gas chromatography. The treated serum samples were filtered through a 0.45 um microporous filter membrane and the content of SCFA was analyzed using a Clarus 680 GAS Chromatograph (PerkinElmer, Boston, MA, USA) [10]. Equipped with a 0.25 μm thick film was used (Elite-FFAP 30 m × 0.25 mm, flow rate 1 mL/min, carrier gas helium). The initial temperature is 100 degrees and the temperature is increased to 180 degrees at 10 °C/min and then increased to 220 °C at 20 °C/min and maintained for 8 min. The temperatures of the injection port and the flame ionization detector were 220 °C and 240 °C, respectively. The helium carrier gas flow rate was 20 mL/min, the airflow rate was 450 mL/min, the hydrogen flow rate was 45 mL/min, the injection volume was 1 uL and the analysis took 18 min. The standards of butyric acid, propionic acid and acetic acid (Sigma Co, St. Louise, MO, USA) were prepared with a concentration of 5 mM, 2 mM and 1 mM.

### 2.9. Next-Generation Sequencing Detection of Gut Microbes

The composition of the gut microbiome was measured from the feces of each mouse and metagene sequencing was performed by using Next generation Sequencing (NGS). DNA from the stool sample was extracted using the QIAamp PowerFecal DNA kit (Mo Bio, Carlsbad, CA, USA) according to the manufacturer’s instructions. According to the method of Jeong et al. [33], the 16 s RNA of DNA in feces was amplified and purified and they were sequenced by Macrogen Ltd. (Seoul, Korea). Mothur (v.1.43.0) was used to process sequence information from fastaq files and filtered according to sequence length >200 bp. Using a MiSeq standard operating procedure, bacteria in each fecal sample were identified, classified and counted. Using Silva’s reference alignment bacterial sequences [33,34], clustering based on high-quality sequences as the operational classification unit (OTU), the 98% similarity was selected for OTU clustering [33].

### 2.10. Statistical Analysis

Statistical analysis was conducted by SPSS version 20.0 (IBM Corp., Armonk, NY, USA). Data are expressed as mean ± standard deviation (SD) or frequency distribution. Significant differences among the groups were determined by one-way analysis of variance (ANOVA) and multiple comparisons among the groups were performed by the Tukey test. The separation of intestinal flora clustering was analyzed by analysis of molecular variance (AMOVA) in Mothur by principal coordinates analysis (PCoA) analysis. *P* < 0.05 was considered a significant difference.

## 3. Results

### 3.1. Total Polyphenol and Flavonoid Contents of STB and AOM Powder and BG Extract

Lyophilized water extracts of STB and AOM contained 68.4 and 41.6 mg gallic acid per g powder for total polyphenols, respectively, whereas they included 14.3 and 8.5 mg rutin per g powder for flavonoids. 30% BG extract of STB and AOM contained 15.2 and 10.3 mg gallic acid per mL extract for total polyphenols and they had 3.6 and 1.9 mg rutin per g powder for flavonoids.

### 3.2. Food, Drug Intake, Body Weight, Fat Weight

The final body weight was lower in the AD-Con than the Norma-Con whereas AD-AOM and AD-MIX retained body weights were similar to the Normal-Con. Since there was no significant difference in initial body weight among the groups, the weight gain during the 5-week experiment was lower in AD-Con and AD-Positive than the Normal-Con and it significantly increased in the ascending order of AD-Positive, AD-Con, AD-STB, AD-AOM, AD-MIX and Normal-Con (*p* < 0.05). However, food intake among the groups was not significantly different among the groups. Food efficiency, calculated by dividing the body weight gain by food intake during the experimental periods, decreased in the AD-Con compared to the Non-AD-Con while AD-AOM and AD-MIX increased food efficiency compared to the AD-CON. The decreased food efficiency was involved possibly in AD severity with increased systemic inflammation and scratching due to itchy. However, AD-Positive decreased food efficiency although AD severity was improved, indicating that other factors might be related to food efficiency (Table 1). Similar to final body weight, AD-Con mice had a lower level of epididymal fat, retroperitoneal fat and total visceral fat than the Normal-Con mice (*p* < 0.05) and their mass in AD-Positive was similar to AD-Con. Total visceral fat mass was higher in AD-AOM and AD-MIX than AD-Con but it was lower than the Normal-Con (*p* > 0.05; Table 1).

### 3.3. The Severity of AD Skin Lesions and Spontaneous Scratching Behavior in AD Mice

**Figure 2:**
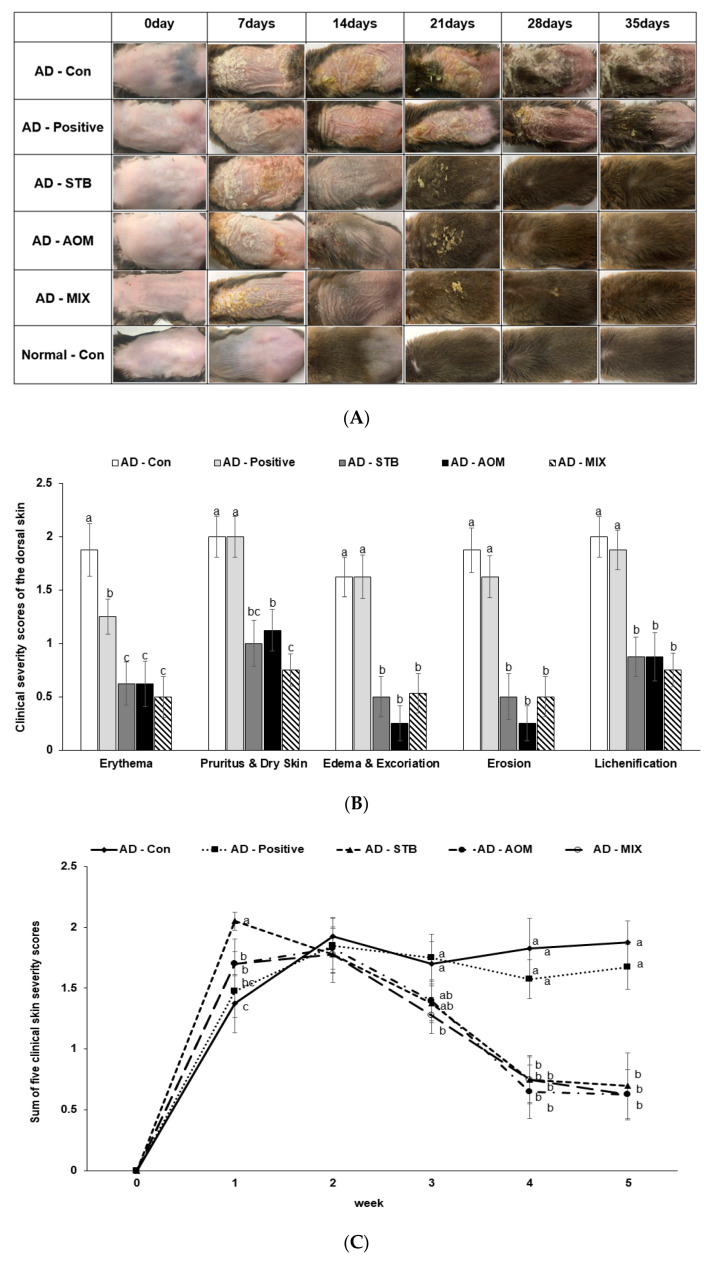

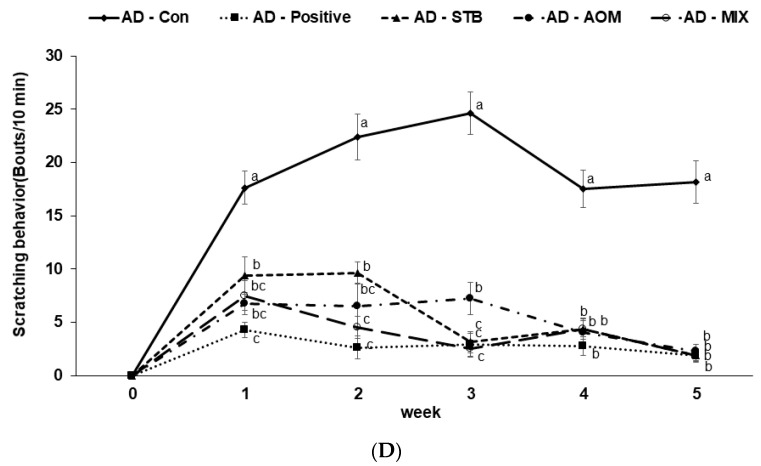
The severity of clinical atopic dermatitis symptoms. (**A**) Changes in atopic dermatitis symptoms at the dorsal skin lesion from week 0 to week 5. (**B**) The severity of each clinical atopic dermatitis symptom at the 5th week (**C**) Weekly scores of an average of 5 clinical atopic dermatitis symptoms from week 0 to week 5. (**D**) Changes in scratching behavior scores from week 0 to week 5. ^a,b^^,c^ Means with different superscripts were significantly different among the groups in each parameter by Tukey test at *p* < 0.05.

In the Normal-Con group, the hair in the dorsal skin grew back within 14 days without any clinical AD symptoms (Figure 2A). After AD induction with DNCB, AD-Con mice induced obvious AD symptoms including erythema, pruritus, dry skin, edema, excoriation, erosion and lichenification (Figure 2A,B). The severity of the clinical symptoms was exacerbated in all AD groups until 7 days and then it was alleviated from day 14 day onward, mainly in the AD-STB, AD-AOM and AD-MIX (Figure 2A,B). The dorsal skin was dry and rough in AD-Positive mice and it was hardened as much as AD-Con but the erythema on the dorsal back was reduced in AD-Positive mice at 14, 21 and 35 days (Figure 2A,B). In the fourth and fifth weeks, the treatment with AD-STB, AD-AOM and AD-MIX alleviated the clinical symptoms in AD mice (*p* < 0.05; Figure 2B). The clinical AD symptoms in the AD-STB and AD-AOM mice were completely alleviated in the 5th week except for having dry hair (Figure 2C). AD-MIX had better-controlled AD clinical symptoms than AD-STB or AD-AOM alone, with no clinical AD symptoms on the skin (Figure 2C).

After 24 h of DNCB application until 3 weeks and without DNCB at the 4th and 5th week, the scratching behavior of mice was visually observed and counted. Compared with the AD-Con, AD-STB, AD-AOM, AD-MIX and AD-Positive treatments reduced the scratching behavior of AD mice (Figure 2D). Scratching behavior was detected at the 4th and 5th weeks in AD-Con but it was reduced in AD-STB, AD-AOM, AD-MIX and AD-Positive although 0.2% DNCB was not applied but had mostly ceased by the 5th week (*p* < 0.05).

### 3.4. Skin Histopathology in AD Mice

**Figure 3:**
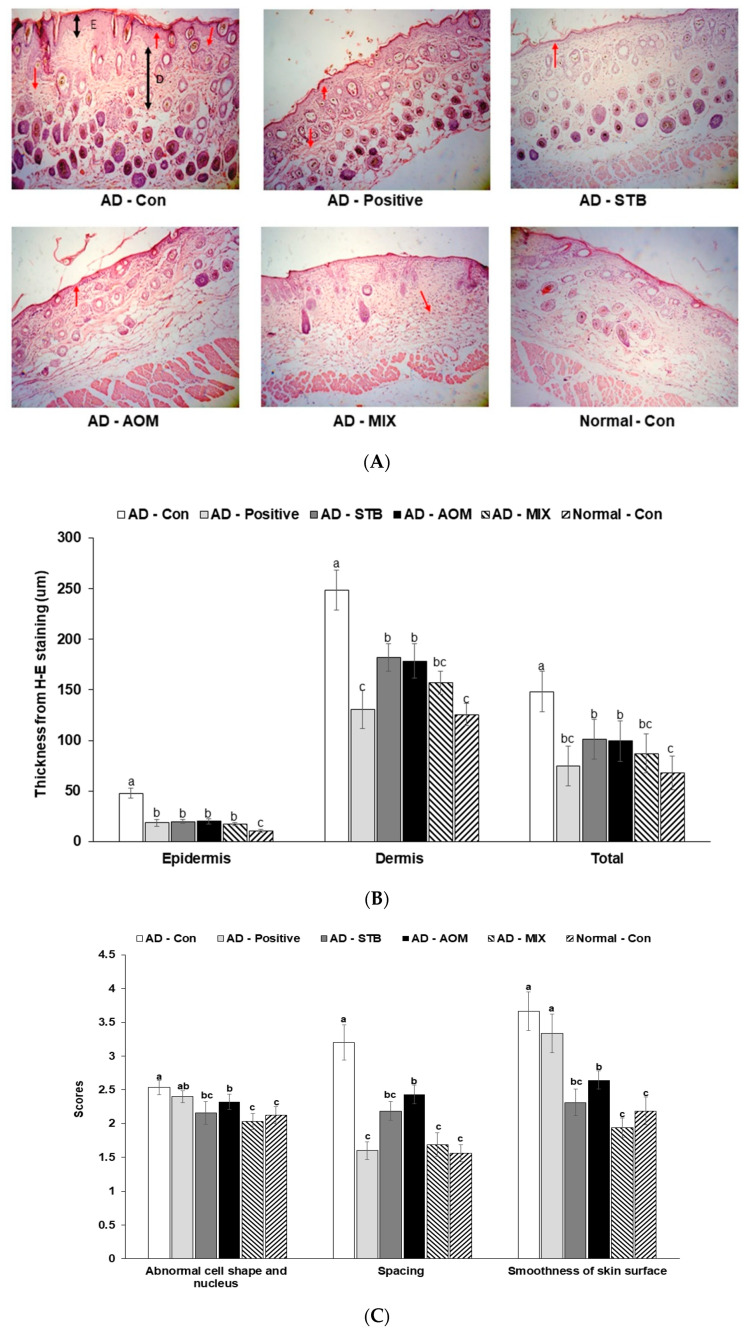

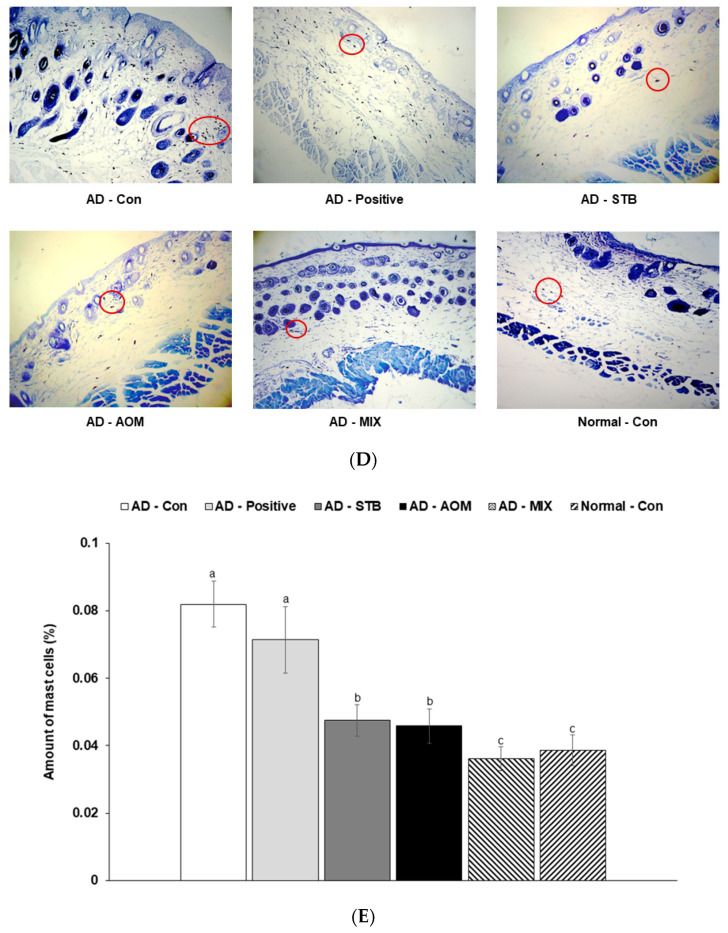
The histological differences and the number of mast cells in the dorsal skin. After the 5-week treatment, the dorsal skin was fixed with 10% formaldehyde, embedded in paraffin and then sections were made. The skin sections were stained with hematoxylin and eosin and toluidine blue staining. The magnification of the image was 100×. (**A**) The histology of the dorsal skin by hematoxylin and eosin staining. (**B**) The thickness of the epidermis (**E**) and dermis (**D**) indicated by blue lines (unit: μm). Total indicated by the sum of epidermis and dermis thickness. (**C**) The abnormality of keratocytes and skin surface indicated by a red arrow. The larger the score, the more abnormal the cell and nucleus shapes, the more spacing between keratocytes and the rougher the skin. (**D**) Mast cell staining by toluidine blue. The mast cells were indicated by the red circles. (**E**) The number of mast cells recognized as blue dots marked by red circles (% of mast cells of the skin tissue area). Each value represents the mean ± SD of 8 mice in each group. ^a,b,c^ Means with different superscripts were significantly different among the groups in each parameter by Tukey test at *p* < 0.05.

In Figure 3A,D, the skin histological changes of AD mice (including epidermal thickening, cell shape and nucleus, spacing, the smoothness of skin surface and mast cells) are shown. In Figure 3B,C,E, AD-Con had thicker epidermis and dermis layers than AD-STB, AD-AOM and AD-MIX and AD-Positive had a similar thickness of epidermis and dermis to AD-STB, AD-AOM and AD-MIX (*p* < 0.05). However, their thickness in AD-STB, AD-AOM and AD-MIX was greater than the Normal-Con. Compared with AD-Con, AD-STB and AD-MIX had better scores for cell shape and nucleus (*p* > 0.05) and the scores in the AD-MIX were similar to the Normal-Con. The spacing of the keratocytes was much higher in the AD-Con than that of Normal-Con, whereas it was reduced in the AD-STB, AD-AOM and AD-MIX groups, compared to the AD-Con (*p* < 0.05). AD-MIX was better keratocyte arrangement than individual treatment of AD-STB and AD-AOM (*p* < 0.05) and AD-MIX showed a similar to the Normal-Con. The roughness of the skin surface was improved in AD-STB, AD-AOM and AD-MIX but it was not enhanced in AD-Positive compared to the AD-Con. The number of mast cells in AD-Con was much higher than the Normal-Con and that was reduced in the AD-STB and AD-AOM and AD-MIX groups compared to the AD-Con (*p* < 0.05). The number of mast cells in the AD-MIX was lowest among the treatment groups (*p* < 0.05) and that in the AD-MIX was similar to that of the Normal-Con. However, the number of mast cells in AD-Positive was similar to AD-Con.

### 3.5. Histopathological Analysis of the Large Intestine in AD Mice

**Figure 4:**
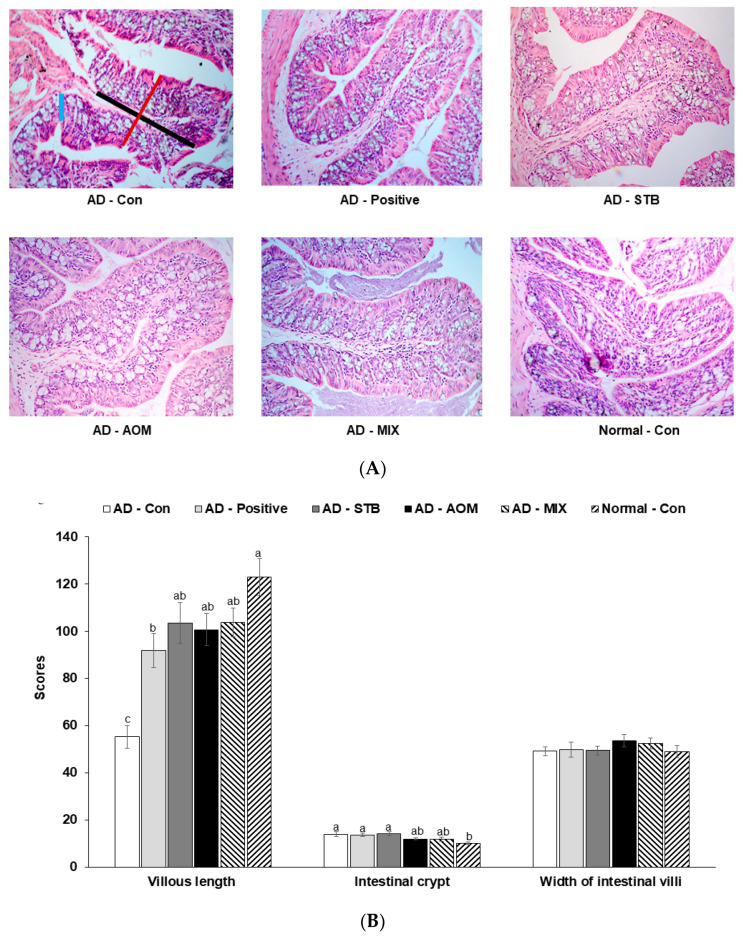

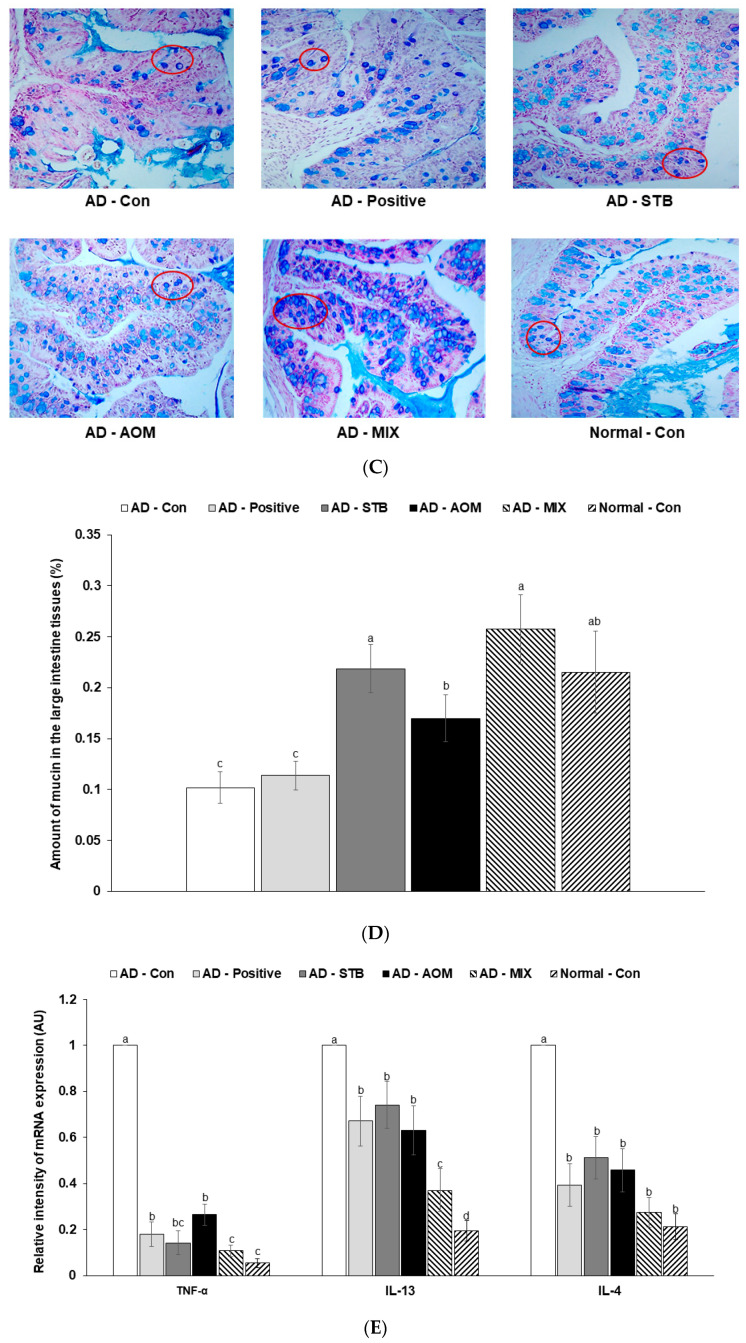
The histological differences and the amounts of mucin in the intestinal tissues among different groups. The intestinal tissues were fixed with 10% formaldehyde, embedded in paraffin and then sections were prepared. The skin sections were stained with hematoxylin and eosin and toluidine blue. The magnification of the image was 200×. (**A**) The histology of the intestinal tissues by hematoxylin and eosin staining. Blueline: Intestinal crypt, Blackline: Length of large intestine villi, Redline: Large intestine villi width. (**B**) The villi length (mm) and inflated and irregular arrangement of the intestinal tissues marked as arrows. The higher scores, the more irregular and inflated intestinal cells. (**C**) The histology of the intestinal tissues by Alcian blue-periodic acid-Schiff staining. (**D**) The percentage of mucin in the large intestinal tissue area. Mucin was marked with red circles in the large intestinal tissues. (**E**) mRNA expression levels of proinflammatory cytokines in the dorsal skin Each value represents the mean ± SD of 5 mice in each group. ^a,b,c^ Means with different superscripts were significantly different among the groups in each parameter by Tukey test at *p* < 0.05.

As shown in Figure 4A, the internal structures of the large intestines in the AD-STB, AD-AOM and AD-MIX groups are complete and clear and the AD-Con large intestine villi are atrophied and damaged. The villous length of the experimental groups was higher than that of AD-Con (*p* < 0.05) and there was no significant difference between the intestinal crypt and width of intestinal villi among the groups of AD mice (Figure 4B). However, the intestinal crypt of the AD-Con was higher than the Normal-Con.

As shown in Figure 4C, mucin was distributed in the intestinal mucosal epithelium and appeared blue with AB-pas staining. The amount of mucin in the AD-Con was lower than Normal-Con. AD-Positive was similar to AD-Con. After the treatment with AD-STB and AD-AOM, the reduction of the mucin amounts in the large intestine in AD mice was improved (*p* < 0.05; Figure 4D).

### 3.6. AD Severity Index and mRNA Expression Levels of Proinflammatory Cytokines in the Dorsal Skin

Serum IgE concentrations were higher in the AD-Con than in other groups (Table 2). AD-Positive treatment did not decrease serum IgE concentrations compared to AD-Con. AD-STB and AD-MIX significantly inhibited the increase of serum IgE concentration compared to AD-Con (*p* < 0.05). The AD-Positive had similar contents of TBARs, a surrogate for lipid peroxide contents, to AD-Con. AD-STB, AD-AOM and AD-MIX lowered TBARs to less than AD-Con but not significantly (*p* > 0.05). Compared with AD-Con, only AD-STB and AD-MIX reduced the content of TBARs in the dorsal skin tissue (*p* < 0.05; Table 2).

The mRNA expression levels of IL-13, IL-4 and TNF-α cytokines in the dorsal skin tissues of AD-Con mice were much higher than the Normal-Con (Figure 4E). Compared with AD-Con, the mRNA expressions of TNF-α in AD-STB, AD-AOM and AD-MIX groups were lower than those in the AD-Con (*p* <0.05; Figure 4E). There were significant differences between AD-Con and Normal-Con in the mRNA expression of IL-13 and IL-4 cytokines (*p* <0.05; Figure 4E). AD-MIX had a similar expression as the Normal-Con. The IL-4 mRNA expressions in the AD-Positive, AD-STB, AD-AOM, AD-MIX and Normal-Con were statistically significantly lower than those in the AD-Con (*p* < 0.05; Figure 4E). The results demonstrated that AD-MIX simultaneously inhibited the expression of TNF-α, IL-13 and IL-4 cytokines, which was better than AD-STB, AD-AOM and AD-Positive.

### 3.7. Liver Damage Index

Serum GOT and GPT levels in the AD-Con group were higher than those in the Normal-Con (Table 2). AD-AOM, AD-STB and AD-MIX showed similar serum GOT and GPT levels to the Normal-Con (*p* < 0.05). Surprisingly, serum GOT and GPT concentrations were much higher in the AD-Positive than the AD-Con. AD-Positive had the highest triglyceride content in the liver (*p* < 0.05) but there were no significant differences among AD-Con and Normal-Con (Table 2). Liver triglyceride deposition was lowered in AD-STB, AD-AOM, AD-MIX than AD-Con (Table 2). These results suggested that AD itself developed liver damage and dexamethasone in the AD-positive exacerbated it whereas STB, AOM and MIX protected against liver damage with reducing inflammation possibly by improving AD symptoms and directly reducing inflammation.

### 3.8. Serum SCFA Concentration

Interestingly, in Table 3, serum acetate concentrations were higher in AD-Con than Normal-Con and the concentrations were decreased in AD-STB, AD-AOM and AD-MIX compared to the AD-Con (*p* < 0.05), with concentrations similar to the Normal-Con. Serum propionate and butyrate concentrations were not significantly different between AD-Con and Normal-Con. However, serum propionate concentrations were higher in AD-AOM and serum butyrate concentrations were higher in AD-STB than other groups (*p* < 0.05). AD-MIX increased serum propionate and butyrate concentrations but not significantly higher than AD-Con (Table 3).

### 3.9. Intestinal Flora

**Figure 5:**
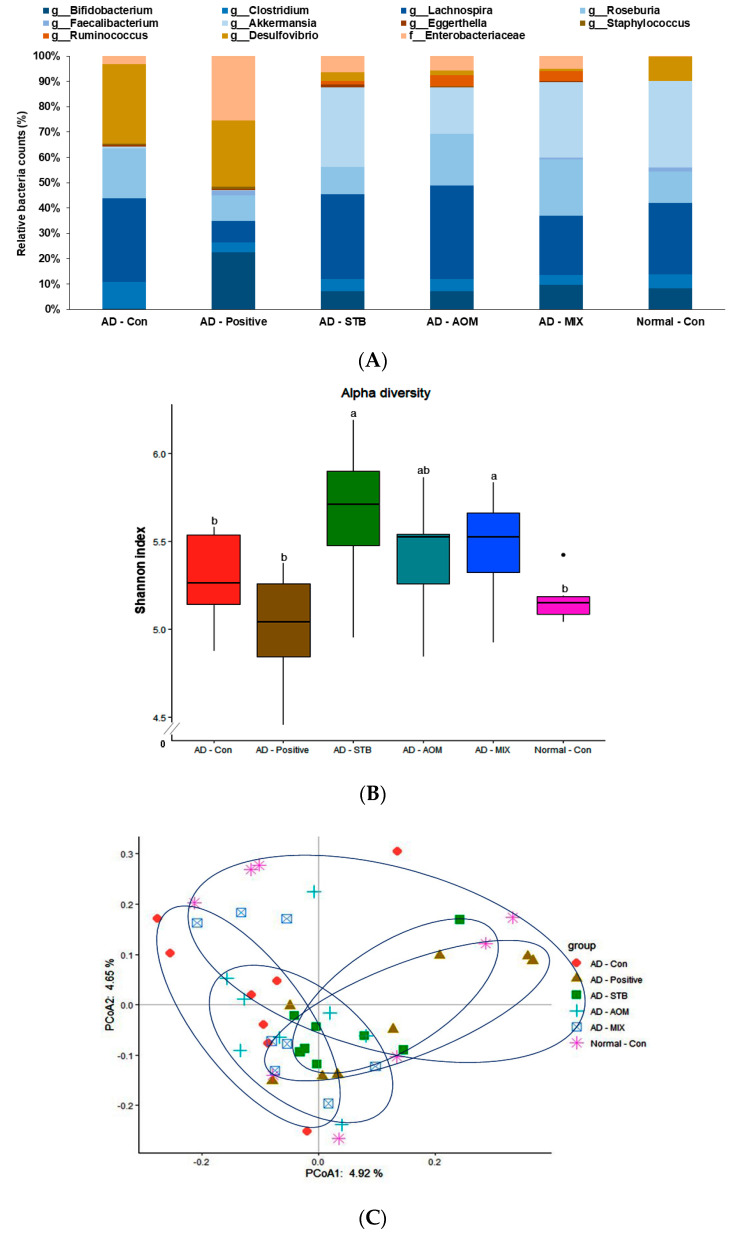
The profiles of gut microbiomes. After 5 weeks of the treatments, feces from the cecum were collected and the bacterial DNA was analyzed by the next generation sequencing method. (**A**) The relative amount (%) of fecal bacteria in the [order] level (**B**) The relative amount (%) of good bacteria for atopic dermatitis in the gut (**C**) The principal coordinate analysis (PCoA) of fecal bacteria. AD-Con vs Positive-C, AD-STB, AD-AOM and AD-MIX of R^2^ were 0.0870 (*p* = 0.016), 0.0863 (*p* = 0.001), 0.0684 (*p* = 0.355) and 0.0700 (*p* = 0.256), respectively. Each value represents the mean ± SD of 5 mice in each group.

At the genus level of the bacteria, a potential effect on atopic dermatitis was determined. *Desulfovibrio*, the harmful bacteria, was higher in the AD-Con group than the Normal-Con group and it was lower in the AD-STB, AD-AOM and AD-MIX groups. *Enterobacteriaceae* was higher in the AD-positive than the other groups (Figure 5A). *Bifidobacterium, Ruminococcus* and *Akkermensia,* beneficial bacteria, were elevated in the AD-STB, AD-AOM and AD-MIX groups, compared to the AD-CON (Figure 5A). *Ruminococcus* and *Akkermensia* were higher in the AD-STB, AD-AOM and AD-MIX groups than the Normal-Con group. An α-diversity is a quantitative procedure to reflect how many different species in a fecal bacteria community. The α-diversity as assessed by the Shannon index was higher in AD-STB and AD-MIX than AD-Con (*p* < 0.05; Figure 5B). A PCoA analysis is used to explore the similarities or dissimilarities of the fecal bacteria species based on the weighted UniFrac distance to their cluster. The bacteria in the AD-Con were separated from those in the AD-Positive and AD-STB (*p* = 0.016 and *p* = 0.001, respectively; Figure 5C). AD-STB and AD-MIX showed a significant separation of fecal bacteria from the AD-Positive at *p* = 0.021 and *p* = 0.03, respectively (Figure 5C). However, other groups had no significant separation in the PCoA analysis. Therefore, AD-STB, AD-AOM and AD-MIX changed the bacteria community and their differences might be associated with the protection against AD.

## 4. Discussion

AD is a chronic recurrent inflammatory skin disease, which can cause severe itching, erythema and edema and other scratching behaviors [27,35]. Recent studies have confirmed that intestinal ecological imbalances are related to changes in immune response and AD. They have shown the effect of intestinal flora on AD due to immune, metabolic and neuroendocrine imbalances [11]. The current commonly used drugs for the treatment of AD are glucocorticosteroids, calcineurin inhibitors, moisturizers and antihistamines, which can improve skin itching, eczema and edema [8,36]. However, long-term usage of some drugs can cause serious side effects such as high blood pressure, heart failure, impaired liver function, skin atrophy and growth retardation in children [8,36]. Alternative medicine including herbal medicine has been studied for treating inflammatory diseases. STB is commonly used for fever, pain relief and anti-inflammatory activities whereas (-)-menthone in STB has analgesic activity and (-)-pulegone in STB has anti-inflammatory activity [17,37]. AOM is a member of the ginger family and is traditionally used for warming the kidney and the spleen and alleviating diarrhea. Buscemi-tertiary compounds are the main components of AOM, which have neuroprotective, anti-aging, anti-inflammatory, anti-allergic effects [20]. Based on these studies, we hypothesized that STB, AOM and STB + AOM may exert an anti-AD effect through enhancing gut microbiome-skin axis in Nc/Nga mice with DNCB applied to the dorsal skin. This current study showed that STB, AOM and STB+AOM improved AD clinical symptoms and suppressed over-activated immunity and pro-inflammatory cytokines, which appear to be linked to improvements in the gut-microbiome-skin axis. The positive-control improved dorsal skin AD symptoms but it did not inhibit the over-activated immunity or enhance the gut microbiome-skin axis and it also induced liver damage as an adverse effect although it was applied to the dorsal skin only.

In the present study, Nc/Nga mice developed human-AD-like clinical symptoms including erythema, pruritus, dry skin, edema, excoriation, erosion and lichenification after the local application of DNCB [27]. DNCB acts as an antigen to induce AD [8,22]. DNCB activates immunity to release IgE from the plasma B cells, similar to the immune defense against parasites but it is associated with over-activating immunity. IgE production results in the degranulation of activating mast cells to release histamine which causes itching and edema of the skin [38]. STB, AOM and the mixture of STB and AOM alleviated the clinical AD symptoms to similar levels as the Normal-Con mice by reducing serum IgE concentrations and mast cells in the skin tissues. The alleviation by STB, AOM and STB+AOM was much better than the AD-positive although dexamethasone was used as an anti-inflammatory drug to treat AD and liver diseases in previous studies [39]. Dexamethasone application on the dorsal skin also improved AD clinical symptoms but it did not reduce serum IgE concentrations and mast cell numbers. This indicated that the local application of dexamethasone sufficiently improved the dorsal skin lesion but it did not result in systemic improvement. Along with serum IgE concentrations, weight gain in the AD-positive was decreased and comparable to the Normal-Con. These results indicate that dexamethasone treatment did not alleviate systemic AD symptoms. Furthermore, dexamethasone increased the activity of GOT and GPT in the serum of the AD-positive group, indicating damage to the liver cells. The contents of triglyceride and TBARs in the liver were abnormally high in the AD-positive compared to AD-Con, although they were higher in the AD-Con than the Normal-Con. AD-STB, AD-AOM, AD-MIX normalized triglyceride and TBARSs to concentrations similar to the Normal-Con. We showed that STB, AOM and STB+AOM protected against liver damage in AD mice.

Chronic atopic dermatitis can cause skin lichenification, cell infiltration and skin thickening [7]. Dexamethasone has an inhibitory effect on the thickening of the epidermis and dermis of AD mice [40]. According to histological observations in the present research, DNCB-induced AD mice showed the thickening of the epidermis and dermis, increased mast cells and eosinophilic granules and severe skin surface damage. AD-STB and AD-AOM, AD-MIX alleviated the thickness and morphological changes of the epidermis and dermis, compared to the AD-Con. In the AD-Positive the thickness of the epidermis and dermis was improved but the morphological changes were not as effectively alleviated as the other treatment groups. These morphological changes have been confirmed to be related to increased mRNA expression levels of skin cytokines [7]. We found that dexamethasone did not effectively inhibit the scratching behavior of AD mice and it was associated with little decrease in the mast cells in the dorsal skin [40]. In another study, pulegone in STB has an inhibitory effect on skin thickness and scratching behavior [37]. Our research indicated that STB and MIX were most effective for alleviating AD induced skin lesions.

AD is a bipolar inflammatory skin disease caused by the imbalance of Th1 and Th2 immune responses [6,41]. As a Th1 cytokine, TNF-α is a key cytokine involved in the pathology of various inflammatory diseases. Its secretion induces the expressions of other cytokines in skin keratinocytes [39]. IL-4 and IL-13 are the key cytokines driving Th2 immune response [6,29]. Patients with AD are well-known to have elevated serum IgE levels, TNF-α, IL-4 and IL-13 [1,4], similar to AD mice in the present research. Although AD-Positive did not reduce the increase of serum IgE, it inhibited the mRNA expression of TNF-α and IL-4 in the dorsal skin as did AD-STB and AD-AOM. AD-STB and AD-AOM improved the skin lesions of AD by reducing mast cells and various cytokines. AD-MIX most effectively inhibited the mRNA expression of TNF-α, IL-4 and IL-13 in the dorsal skin and reduced the number of mast cells. These results indicated that AD-MIX effectively treated AD symptoms by regulating the balance of Th1 and Th2. The combination of AD-STB and AD-AOM is an effective intervention for the treatment of AD.

In recent years, many studies have focused on the relationship between gut microbes, short-chain fatty acids and AD [9,12,13]. Our study showed that AD mice increased serum acetate concentrations among SCFA and compared with the AD-Con, only AD-STB and AD-MIX reduced serum acetic acid concentrations and increased butyric acids. Also, the bacteria in the intestines, including large intestines, reflects the body’s immune activity to a certain extent [42]. The increase in the length of the large intestinal villi can enhance the digestion and absorption of food and the height of the large intestine villi and the depth of the intestinal crypt are known indicators of intestinal function and intestinal health [31]. Goblet cells of the intestinal mucosal epithelium secrete mucin, forming an intestinal mucosal epithelial barrier to separate intestinal bacteria and epithelial cells [43]. The balance of the mucin barriers plays an important role in the mutual symbiosis between many intestinal commensal bacteria and the host, suggesting that mucin modulation is an important factor for the intestinal ecological balance [43]. In the AD mice, the large intestine had shorter villi and a lesser amount of mucin, which disturbed the integrity of the intestinal epithelium. Dexamethasone treatment did not improve the intestinal morphology in AD mice. STB and STB+AOM improved the balance of the microflora in the large intestines of AD mice and increased the length of the villi and mucin content to regulate the intestinal health. Our research suggested that STB and STB+AOM were more effective for promoting intestinal health in AD mice.

In addition to changes in the intestinal morphology, there are differences in the composition of intestinal flora between AD patients and healthy people [9]. There is an internal relationship between the skin and the intestinal flora [9]. Our research explored the effects of AD-Positive, AD-STB, AD-AOM and AD-MIX on the intestinal bacteria of AD mice and was also tested through the NGS platform. *Clostridium* and *Escherichia coli* in intestinal microbes of AD patients are higher than those of healthy people and *Bacteroidetes* and *Bifidobacteria* are be reduced [5]. The Shannon index, α-diversity index, is reported to be related to the intestinal health and also influences the immunity of the host [44]. The present study confirmed the relationship between dietary intake of AD patients, intestinal flora and the skin immune system. The analysis found that, compared with the control group, AD-STB and AD-MIX increased the α-diversity index indicating the intestinal microbial community richness and community diversity. PCoA clustering analysis showed the differences in intestinal bacteria composition among the groups and AD-STB showed a clear separation of gut bacteria from the AD-Con (*p* = 0.001).

## 5. Conclusions

Simultaneous treatment of STB, AOM and MIX in the oral intake and local application can effectively treat the clinical symptoms of AD mice by improving Th1/Th2 balance and reducing pro-inflammatory cytokines through skin anti-inflammatory direct effect and an indirect effect by the gut microbiome changes. Mainly their intake enhanced intestinal immunity by gut microbiome-skin axis through modulating serum SFCA concentration and improved the diversity of intestinal bacteria and intestinal tissues in AD mice. AD-MIX had an alleviation of AD symptoms at best without adverse effects. These results showed that STB and AOM herbal extracts had pharmaceutical activities for AD treatment although it needs to further exert better efficacy in humans.

## Figures and Tables

**Table 1:** Body composition and food intake at the end of experimental periods.

Metabolic Parameters	AD-Con	AD-Positive	AD-STB	AD-AOM	AD-MIX	Normal-Con
Final weight (g)	22.6 ± 1.29 ^ab^	19.3 ± 1.95 ^b^	24.6 ± 1.32 ^ab^	24.9 ± 2.76 ^ab^	25.1 ± 1.75 ^a^	27.3 ± 1.70 ^a^
Weight gain (g)	1.94 ± 1.03 ^c^	0.31 ± 1.22 ^c^	3.44 ± 0.48 ^bc^	5.81 ± 1.28 ^ab^	5.43 ± 0.97 ^ab^	8.43 ± 1.27 ^a^
Food intake (g/day)	3.37 ± 0.17	2.89 ± 0.15	3.39 ± 0.26	3.34 ± 0.16	3.46 ± 0.17	2.99 ± 0.16
Efficiency of food	0.56 ± 0.31 ^c^	0.11 ± 0.42 ^c^	1.02 ± 0.14 ^bc^	1.82 ± 0.46 ^ab^	1.64 ± 0.35 ^b^	2.82 ± 0.38 ^a^
Epididymal fat (g)	0.60 ± 0.16 ^b^	0.39 ± 0.16 ^b^	0.73 ± 0.11 ^b^	0.93 ± 0.26 ^ab^	0.86 ± 0.19 ^b^	1.40 ± 0.17 ^a^
Retroperitoneal fat (g)	0.23 ± 0.07 ^b^	0.15 ± 0.07 ^b^	0.28 ± 0.05 ^ab^	0.31 ± 0.08 ^ab^	0.32 ± 0.08 ^ab^	0.48 ± 0.08 ^a^
Total visceral fat (g)	0.83 ± 0.23 ^b^	0.54 ± 0.23 ^b^	1.01 ± 0.15 ^b^	1.25 ± 0.34 ^ab^	1.19 ± 0.27 ^ab^	1.88 ± 0.25 ^a^

No atopic dermatitis induced mice in the Normal-CON without the application of DNCB in the dorsal skin (30% BG). The DNCB applied Nc/Nga mice had the dorsal skin topical application of 30% water, 0.01% dexamethasone, 30% STB, 30% AOM, 15% STB + 15% AOM in BG extract plus 1% dextrin, 1% STB, 1% AOM and 0.5% STB + 0.5% AOM (AD-Con, AD-Positive, AD-STB, AD-AOM and AD-MIX) in high-fat diets, respectively. The efficiency of food is obtained by dividing the weight gain by the food intake. Values are entered as mean ± SD (*N* = 8). Different letters indicate a significant difference (*p* < 0.05).

**Table 2:** Index of atopic dermatitis-related parameters and liver damage.

Metabolic Parameters	AD-Con	AD-Positive	AD-STB	AD-AOM	AD-MIX	Normal-Con
Serum IgE (mg/dl)	3121 ± 397 ^a^	2662 ± 481 ^ab^	1397 ± 328 ^c^	1679 ± 183 ^bc^	1335 ± 251 ^c^	1380 ± 174 ^c^
Skin TBARs (mg/dl)	13.5 ± 1.72 ^ab^	15.0 ± 1.88 ^a^	7.6 ± 1.26 ^bc^	9.4 ± 1.45 ^abc^	6.6 ± 0.82 ^bc^	4.4 ± 0.91 ^c^
Serum GOT (mg/dl)	58.3 ± 7.56 ^b^	98.6 ± 16.93 ^a^	30.7 ± 3.07 ^c^	42.7 ± 5.57 ^bc^	32.6 ± 2.44 ^c^	33.5 ± 1.44 ^c^
Serum GPT (mg/dl)	10.5 ± 4.93 ^ab^	14.4 ± 4.07 ^a^	1.7 ± 2.67 ^b^	5.9 ± 4.04 ^ab^	1.7 ± 2.10 ^b^	1.8 ± 2.17 ^b^
Liver Triglyceride (mg/g tissue)	2.77 ± 1.01 ^bc^	7.39 ± 1.81 ^a^	0.98 ± 0.29 ^c^	1.66 ± 0.65 ^c^	1.65 ± 0.46 ^c^	3.28 ± 0.60 ^b^

No atopic dermatitis induced mice in the Normal-Con without the application of DNCB in the dorsal skin (30% BG). The DNCB applied Nc/Nga mice had the dorsal skin topical application of 30% water, 0.01% dexamethasone, 30% STB, 30% AOM, 15% STB + 15% AOM in BG extract plus 1% dextrin, 1% STB, 1% AOM and 0.5% STB + 0.5% AOM (AD-Con, AD-Positive, AD-STB, AD-AOM and AD-MIX) in high-fat diets, respectively. The efficiency of food is obtained by dividing the weight gain by the food intake. Values are entered as mean ± SD (*N* = 8). Different letters indicate a significant difference (*p* < 0.05).

**Table 3:** Short-chain fatty acids (SCFA) concentration in portal venous serum.

SCFA	AD-Con	AD-Positive	AD-STB	AD-AOM	AD-MIX	Normal-Con
Acetic acid (mM)	0.107 ± 0.017 ^a^	0.102 ± 0.012 ^a^	0.077 ± 0.001 ^b^	0.080 ± 0.001 ^b^	0.079 ± 0.002 ^b^	0.086 ± 0.03 ^b^
Propionic acid (mM)	0.034 ± 0.001 ^b^	0.036 ± 0.003 ^b^	0.043 ± 0.001 ^a,b^	0.054 ± 0.002 ^a^	0.043 ± 0.0005 ^a,b^	0.041 ± 0.005 ^a,b^
Butyric acid (mM)	0.028 ± 0.001 ^b^	0.027 ± 0.001 ^b^	0.041 ± 0.007 ^a^	0.027 ± 0.001 ^b^	0.038 ± 0.002 ^a,b^	0.031 ± 0.001 ^b^
Total (mM)	0.170 ± 0.020	0.165 ± 0.015	0.161 ± 0.009	0.161 ± 0.022	0.160 ± 0.004	0.158 ± 0.011

No atopic dermatitis induced mice in the Normal-Con without the application of DNCB in the dorsal skin (30% BG). The DNCB applied Nc/Nga mice had the dorsal skin topical application of 30% water, 0.01% dexamethasone, 30% STB, 30% AOM, 15% STB + 15% AOM in BG extract plus 1% dextrin, 1% STB, 1% AOM and 0.5% STB + 0.5% AOM (AD-Con, AD-Positive, AD-STB, AD-AOM and AD-MIX) in high-fat diets, respectively. Values are entered as mean ± SD (*N* = 8). Different letters indicate a significant difference (*p* < 0.05).
